# Optimization of Gate-All-Around Device to Achieve High Performance and Low Power with Low Substrate Leakage

**DOI:** 10.3390/nano12040591

**Published:** 2022-02-09

**Authors:** Changhyun Yoo, Jeesoo Chang, Sugil Park, Hyungyeong Kim, Jongwook Jeon

**Affiliations:** 1 Department of Electrical and Electronics Engineering, Konkuk University, Seoul 05029, Korea; hero0056@konkuk.ac.kr (C.Y.); tnrlf99@konkuk.ac.kr (S.P.); khgyung98@konkuk.ac.kr (H.K.); 2 Data and Information Tech. (DIT) Center, Samsung Electronics, Hwasung-Si 18448, Korea; jess907@naver.com

**Keywords:** multi-nanosheet FET, punch-through-stopper, bottom oxide, ring oscillator, SRAM, full adder

## Abstract

In this study on multi-nanosheet field-effect transistor (mNS-FET)—one of the gate-all-around FETs (GAAFET) in the 3 nm technology node dimension—3D TCAD (technology computer-aided design) was used to attain optimally reduced substrate leakage from options including a punch-through-stopper (PTS) doping scheme and a bottom oxide (BO) scheme for bottom isolation, with the performance improvement being shown in the circuit-level dynamic operation using the mNS-FET. The PTS doping concentration requires a high value of >5 × 10^18^ cm^−3^ to reduce gate induced drain leakage (GIDL), regardless of the presence or absence of the bottom isolation layer. When the bottom isolation is applied together with the PTS doping scheme, the capacitance reduction is larger than the on-state current reduction, as compared to when only the PTS doping concentration is applied. The effects of such transistor characteristics on the performance and capabilities of various circuit types—such as an inverter ring oscillator (RO), a full adder (FA) circuit, and a static random-access memory (SRAM)—were assessed. For the RO, applying BO along with the PTS doping allows the operating speed to be increased by 11.3% at the same power, or alternatively enables 26.4% less power consumption at the same speed. For the FA, power can be reduced by 6.45%, energy delay product (EDP) by 21.4%, and delay by 16.8% at the same standby power when BO and PTS are both applied. Finally, for the SRAM, read current (I_READ_) increased by 18.7% and bit-line write margin (BWRM) increased by 12.5% at the same standby power. Through the circuit simulations, the Case 5 model (PTS doping concentration: 5.1 × 10^18^ cm^−3^, with BO) is the optimum condition for the best device and circuit performance. These observations confirm that PTS and bottom isolation applications in mNS-FETs can be utilized to enable the superior characteristics of such transistors to translate into high performance integrated circuits.

## 1. Introduction

Planar metal-oxide semiconductor field-effect transistors (MOSFETs) with single gates have scaled down over a long period of time according to Moore’s law and Dennard’s scaling rule. The increasing prominence of short channel effects (SCE) with reductions in MOSFET size, however, have resulted in the fin field-effect transistor (FinFET) being introduced to enable further reductions in transistor size. The FinFET structure, introduced near the 20 nm technology node for semiconductor device fabrication, has been successfully applied up to the (most recent past) 5 nm technology node. The fin channel became thinner to suppress SCE, which became more severe as scaling down went further. In addition, the fin height was gradually increased to obtain a larger effective width in the same footprint. These methods of intensifying the FinFET design are also close to their limits, however, such that multi-nanosheet field-effect transistor (mNS-FETs) will be used near the 3 nm technology node [1,2]. Using gate-all-around (GAA) type nanosheet channels stacked in multiple layers, mNS-FET has a wider effective width in the same footprint and good gate controllability characteristics compared to Fin-FET, so it is understood that mNS-FET can be applied to a more scaled-down technology node [3].

**Figure 1:**
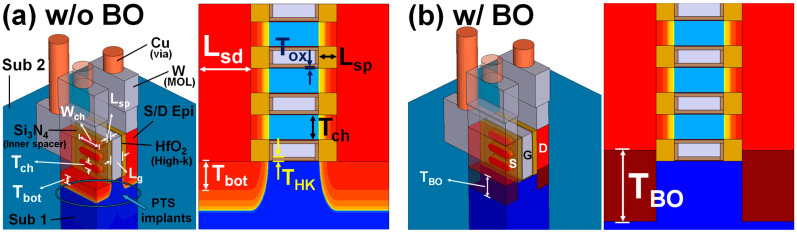
(**a**) 3D structure and cross section view of mNS-FET with PTS doping scheme, (**b**) 3D structure and cross section view of mNS-FET with bottom isolation scheme.

An issue that is requiring attention in mNS-FET development is that of leakage current. As shown in Figure 1, when three nanosheets are used in the mNS-FET structure, a channel is also created in the substrate by the bottom gate (in addition to the nanosheet), which is a structure similar to a planar MOSFET. Such a channel created in the lower substrate results in a high risk of leakage current in the 3 nm dimension. Ideas discussed to date to reduce such substrate leakage include: (i) inserting an insulating oxide layer for bottom isolation, which is referred to here as then bottom oxide (BO) scheme [4,5] and (ii) doping the bottom substrate, which is referred to as the punch-through-stopper (PTS) doping scheme [6,7]. While the PTS doping scheme and the bottom oxide (BO) scheme can be studied separately, there has been no analysis of simultaneous optimization to date. Therfore, this gap in the analysis is addressed in this study.

In our previous study on BO thickness based on the presence or absence of BO application and substrate [8], we confirmed that BO successfully reduced the leakage current. As an extension of that previous study, the current study tried to optimize PTS doping and BO at the same time; since PTS doping and BO application include capacitance-voltage (C-V) characteristics in addition to current-voltage (I-V) characteristics, the effect of reducing leakage current on various logic/memory circuits was investigated. In this study, for the first time, device optimization and characteristics analysis were performed in various circuits considering both PTS doping and BO. We believe that the results of this study will become guidelines for substrate engineering that enable leakage current to be reduced and optimal circuit performance to be achieved when designing 3 nm or smaller mNS-FET devices.

The remainder of this paper proceeds as follows. First, the 3D-TCAD and circuit simulation environment used in this study are explained, and the mNS-FET device characteristics are described for six main cases in which PTS doping and BO characteristics are changed. Second, the effects of PTS doping and BO characteristics on the operating speed and power consumption of a four-bit full adder (FA) operation circuit composed of basic inverter ring oscillator (INV RO) and various standard cells are examined. In addition, the noise margin characteristics in the reading and writing of the static random-access memory (SRAM) circuit are examined. Finally, we conclude and summarize the results of the analysis.

## 2. mNS-FET Device Optimization and Circuit Analysis

Figure 1 shows the mNS-FET structure in the 3 nm technology node dimension, where Figure 1a,b show the structure in which only PTS doping is applied, and in which PTS doping and BO are applied together, respectively. The contact poly pitch (CPP), channel length (Lch), and effective oxide thickness (EOT), which are key dimensions in 3 nm technology node, were taken from IRDS 2020 [9]. The nanosheet width and the number of nanosheets in a single stack were taken from a study dealing with the optimal design of the existing mNS-FET [1]. The device parameters used in the simulation are listed in Table 1.

**Figure 2:**
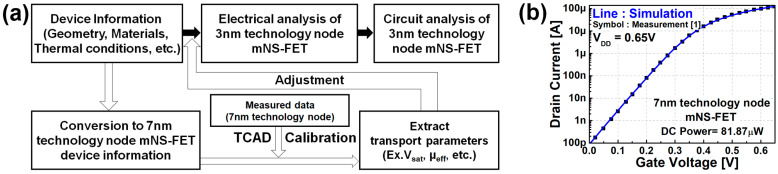
(**a**) Overall workflow for the analysis of mNS-FET, (**b**) I_DS_ versus V_GS_ calibration result based on the measurement of a fabricated mNS-FET.

Synopsys Sentaurus (Synopsys Inc., Mountain View, CA, USA), a commercial 3D technology computer-aided design (TCAD) software package, was used for the analysis, with a drift-diffusion model of electrical transport being used for the analysis. The overall workflow of this study and the calibration of the model used in the simulations are shown in Figure 2.

To increase the accuracy of the simulation, calibration of the mobility model and velocity saturation model parameters used in the simulator was carried out with the I-V characteristics measured from mNS-FET hardware manufactured in the 7 nm technology node dimension. The carrier transport model used for the calibration is as follows:

- To predict the subthreshold behavior accurately and apply the doping/temperature dependence, the Shockley–Read–Hall (SRH) recombination model (available in Synopsys Sentaurus Device) was included.
- The density gradient quantization model (eQuantumPotential) was included to describe the quantum confinement effect.
- The mobility model (PhuMob + High Field Saturation + Enormal) was utilized to consider the quantum effect, Coulomb scattering, and interfacial surface roughness scattering [10].
- The Lombardi mobility model was included to calculate the mobility degradation by remote phonon and Coulomb scatterings at the channel and insulator interface [11].
- A thin-layer mobility model was used to account for the thin channel thickness.

Using the results of this calibration, the mNS-FET characteristics in the 3 nm technology node dimension of this study were predicted. In addition, the off-state current (I_OFF_, which is the drain-source current (I_DS_) value under the gate-source and drain-source voltage conditions of V_GS_ = 0 V and V_DS_ = V_DD_ (drain supply voltage)) was targeted to 100 pA by adjusting the work function of the gate metal, assuming a replacement metal gate process; 100 pA is appropriate for low-power applications. The model library for circuit simulation was developed using the industry standard compact model BSIM-CMG (ver. 110.0) [12] from the I-V and C-V characteristic curves, which are the device level analysis results. In this case, it is assumed that the positive-channel metal-oxide (PMOS) has symmetrical characteristics with the negative-channel metal-oxide (NMOS) analyzed as TCAD.

**Figure 3:**
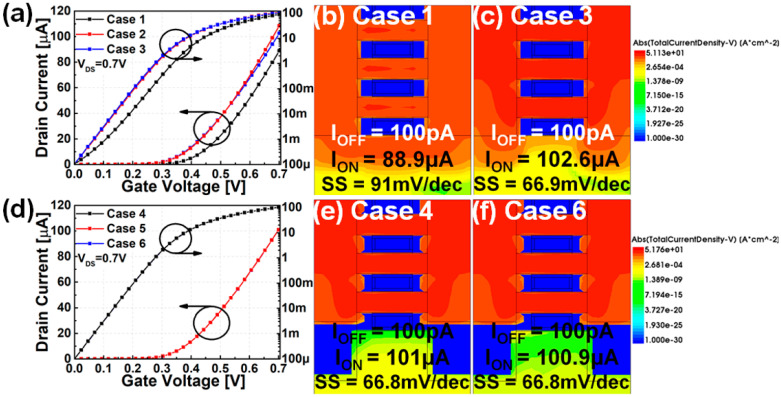
(**a**) I_DS_ versus V_GS_ characteristics for cases 1–3, Current density profile for (**b**) Case 1, (**c**) Case 3, (**d**) I_DS_ versus V_GS_ characteristics for cases 4–6, Current density profile for (**e**) Case 4, (**f**) Case 6.

Figure 3 shows the transfer curve and current density of the mNS-FET according to the presence or absence of PTS doping and BO, which was the basis for classifying cases in this study. Ambient temperature was considered as 300 K in the device and circuit simulations. Cases 1–3 are labels for cases that used the without-BO model, and Cases 4–6 are those that used the with-BO model. As shown in Figure 3a, the I_ON_/I_OFF_ ratio and sub-threshold swing (SS) values change according to the difference in the PTS doping concentration (N_SUB,PTS_). Figure 3b shows that the substrate channel seen below the bottom gate becomes the largest leakage path, and as shown in Figure 3c, it can be seen that increasing N_SUB,PTS_ improves the I_ON_/I_OFF_ and SS values. However, in the substrate region with a very short channel such as a 3 nm technology node, the high doping concentration results in a high risk of widening the process variation between devices, so there is a trade-off between performance and electrical characteristics variation.

Figure 3d shows the variation of I_ON_/I_OFF_ ratio and SS value of mNS-FET applying BO. Figure 3e–f show that the substrate leakage path under the bottom gate is properly blocked even at low N_SUB,PTS_ in the case of applying BO, so electrostatic characteristics similar to or better than those of highly doped N_SUB,PTS_ can be confirmed. In addition, there is no significant difference in the I-V characteristics depending on the N_SUB,PTS_. These results imply that BO can be used to avoid highly doped N_SUB_,_PTS_, which increases the process variation.

**Figure 4:**
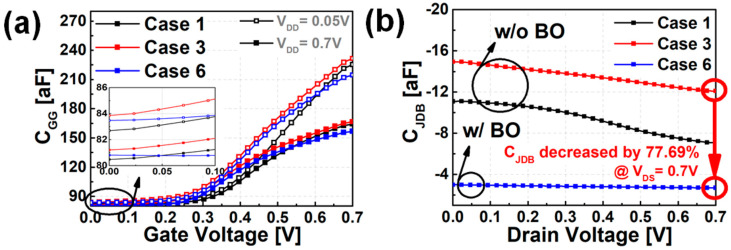
(**a**) PTS doping concentration versus C_GG_ for case 1, 3, 6, (**b**) C_JDB_ versus drain voltage for case 1, 3, 6.

Figure 4 shows the C-V characteristics of the mNS-FET according to PTS doping and the presence/absence of BO. When the same PTS doping is applied, the parasitic capacitance component (=C_GGOFF_, C_JDB_) in the off-state is smaller when bottom isolation is applied. As shown in Figure 4a–b, if BO is applied, the doping concentration of the bottom substrate can be lightly doped, so the widened depletion width and the overlap section between the gate and the source/drain disappear, so C_GGOFF_ is reduced. In addition, as shown in Figure 4b, the junction capacitance (C_JDB_) caused by the depletion region between the source/drain and the substrate is screened out owing to the BO, resulting in additional parasitic capacitor reduction.

**Figure 5:**
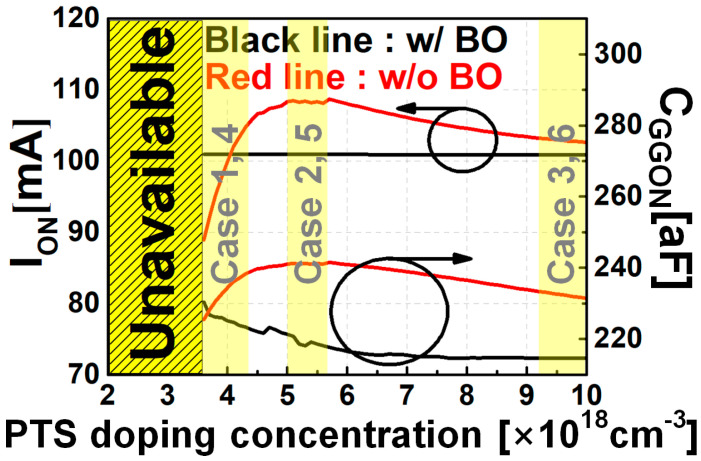
PTS doping concentration versus I_ON_/C_GGON_ for the presence or absence of BO.

The PTS doping concentration ranges are shown in Figure 5. Figure 5 summarizes the current (I_ON_) and capacitance (C_GGON_) characteristics in the ON state after I_OFF_ targeting. As shown in Figure 5, when BO does not exist, the optimal point of I_ON_ exists according to N_SUB,PTS_. When BO exists, there is no change in I_ON_ according to N_SUB,PTS_. When the N_SUB,PTS_ is approximately 3.2 × 10^18^ cm^−3^ or less, the substrate current becomes too large because of gate-induced barrier lowering(GIDL), so it is difficult to satisfy the static leakage power specification. If BO does not exist, as the N_SUB,PTS_ decreases, the C_GGON_ value is determined by the concurrent actions of two phenomena: C_GGON_ would increase because of the threshold voltage(V_TH_) shift for I_OFF_ targeting, but C_GGON_ would also decrease due to depletion charge between source/drain and channel. In the case of there being BO, as the latter mechanism disappears, we expect to see C_GGON_ increase as N_SUB,PTS_ decreases.

Circuit operation characteristics affect not only direct current (DC) I-V characteristics, but also alternating current (AC) C-V characteristics. As shown in Figure 5, the BSIM-CMG model library, which can accurately describe the I-V and C-V characteristics of mNS-FETs in six cases according to the BO and PTS doping process, was developed.

**Figure 6:**
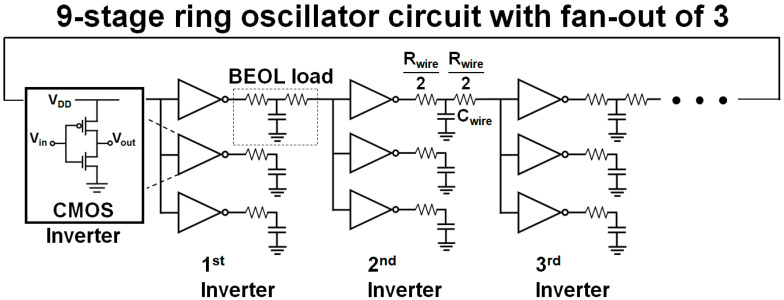
Nine stages RO schematic with fan-out of three.

The ring oscillator (RO) used for this benchmark is an inverter (INV) chain consisting of nine stages and a fan-out of 3 (FO3). Figure 6 shows the circuit. The interconnect characteristics considered in this work are middle-end-of-line (MEOL) resistance/capacitance, back-end-of-line (BEOL) resistance/capacitance, and contact resistivity, which are 105 Ω, 9.96 aF, 301 Ω/μm, 208 aF/μm, and 1 × 10^−9^ Ω∙cm^−2^, respectively [9,13]. It is assumed that the NMOS/PMOS used in the circuit configuration are in balance with each other. Because all six cases have the same I_OFF_ value, the static leakage power of the circuit is also the same. 

**Figure 7:**
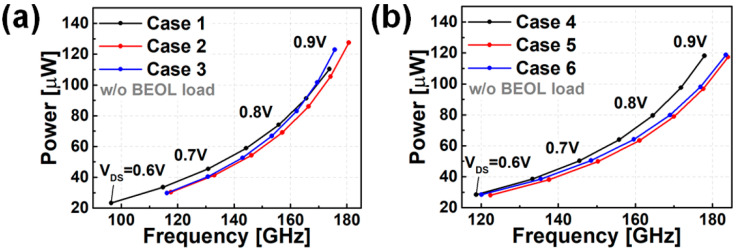
PPA results of nine stages RO (without BEOL) for (**a**) without BO models (case 1–3), (**b**) with BO models (case 4–6).

**Figure 8:**
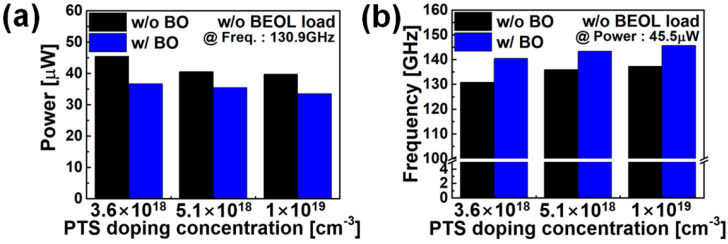
PPA summaries of nine stages RO in condition of (**a**) iso-speed (**b**) iso-power.

Figure 7 shows the results of observing the active power and operating frequency of FO3 INV RO while changing the supply voltage (V_DD_). Figure 7a shows the circuit characteristics when only the PTS doping scheme is applied. Depending on N_PTS,SUB_, it can be seen that the speed can be increased by 3.9~4.9% under the iso-power condition, and the power can be decreased by 10.8~12.6% under the iso-speed condition (where comparison is necessary under the V_DD_ = 0.7 V condition). Figure 7b shows the circuit characteristics according to the change in the PTS doping scheme in the state where BO is applied. It can be confirmed that the speed and power characteristics are improved by applying BO. Compared to the case where only the PTS doping scheme is applied, the speed is improved by 2% to 3.7% in the iso-power condition when BO is also applied, and the power is reduced by 3.5–8.7% in the iso-speed condition. Even when BO is applied, there is a change in the circuit characteristics (speed ~3.7%, power ~8.7%) depending on the PTS doping. Therefore, even if BO is applied, the optimization design of PTS doping is necessary. The circuit benchmark summaries are presented in Figure 8.

**Figure 9:**
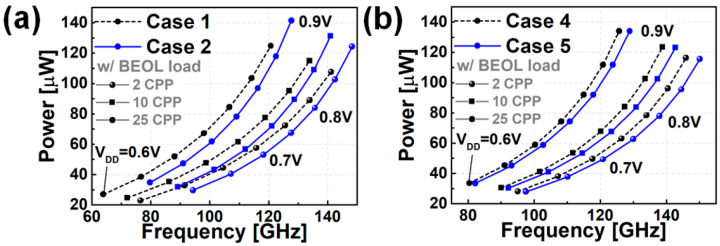
PPA results of nine stages RO (with BEOL load) for (**a**) without BO models (case 1 and 2), (**b**) with BO models (case 4 and 5).

Figure 9 shows the circuit characteristics when the BEOL load is considered. As the distance between devices increases, the BEOL load value increases, and the degradation of speed and power characteristics worsens. Figure 9a shows the PPA results for Cases 1 and 2, in which BO is not applied. When the length of the BEOL increases from 2CPP to 25CPP, in Case 1, it decreases by 9.7% and 17% under iso-power conditions (the power of 2CPP RO when V_DD_ = 0.7 V) and increases by 23% and 45% under iso-speed conditions (the speed of 2CPP RO when V_DD_ = 0.7 V). Figure 9b shows the power, performance, area (PPA) results of Cases 4 and 5 to which BO is applied; when the length of BEOL increases from 2CPP to 25CPP, in Case 4, it decreases by 7.4% and 13.4% under iso-power conditions and increases by 27.8% and 59.6% under iso-speed conditions. When BO is applied, the effective capacitance (C_EFF_; sum of C_GGON_, C_JDB_, and C_FO_ (fanout capacitance)) of INV is reduced, which affects the RO delay. The simulation results showed that the speed degradation in the iso-power condition was lowered according to the length of the BEOL in the model to which BO was applied. Therefore, it can be expected that the tolerance to degradation caused by BEOL on the circuit is increased as a result of BO.

**Figure 10:**
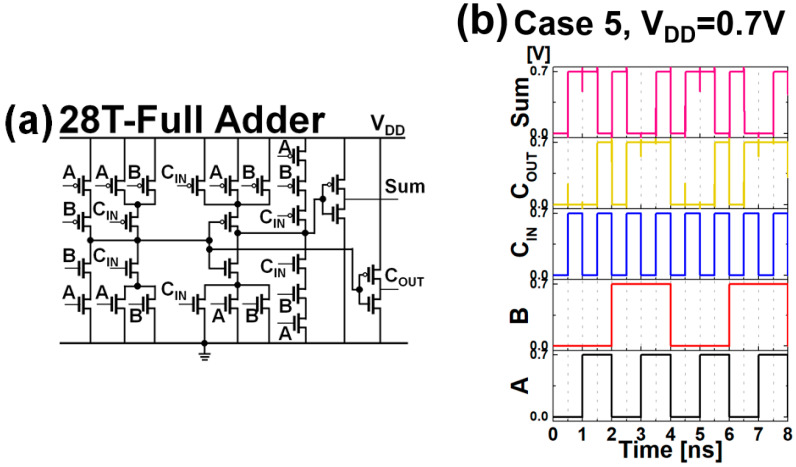
(**a**) Conventional 28T-FA schematic, (**b**) input/output versus time for case 5 when the V_DD_ is 0.7 V.

The 28T-full adder (FA) was designed using mNS-FETs, and the performance optimization according to PTS doping and BO was analyzed with key figures of merit (FoM): dynamic power (P_dyn_), energy-delay product (EDP), and delay. Figure 10a shows a conventional 28-T FA circuit designed with 14 N/P-type mNS-FETs. Figure 10b shows the operation of the 1-bit FA and two cycles of output from input 000 to 111. In this study, P_dyn_ was measured using the average current of the operating FA circuit (I_AVG_ × V_DD_), and P_stat_ was measured as I_OFF_ × V_DD_. In a logic circuit, the power-delay product (PDP) is an indicator of the energy efficiency of a logic gate. EDP is a value obtained by dividing PDP by clock frequency to compare the PDP at the same clock frequency. EDP is expressed as the product of energy and delay: the lower the value, the better the efficiency. A four-bit FA was used to obtain the delay [14]. When ‘0000 + 1111 + 1’ is calculated, we measure the time taken from the first carry-in (C_IN_) to the calculation of the carry-out (C_OUT_) of the last four-stage full adder. From the simulation exercise, P_dyn_ of Case 2 has the highest value, which seems to have affected the results because I_ON_ of Case 2 was the highest. Similar to RO, in FA, the circuit performance of Case 5 shows the best performance in terms of power and speed. Comparing Case 5 and Case 2, P_dyn_ of Case 5 decreased by approximately 15% and delay by 5.6%. The overall FA simulation results are listed in Table 2.

**Figure 11:**
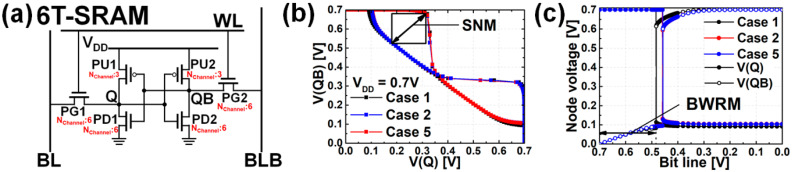
(**a**) 6T-SRAM schematic, (**b**) SNM results when the V_DD_ is 0.7 V, (**c**) BWRM results when the V_DD_ is 0.7 V.

Static random-access memory (SRAM) was designed using mNS-FETs, and the performance optimization according to PTS doping and BO was analyzed with key figures of merit (FoM): static noise margin (SNM), read current (I_READ_), and bit-line write margin (BWRM). Figure 11a shows a 6T-SRAM cell, and the ratio of the effective channel widths (W_EFF_) of the pull-up (Figure 11a in PU), pass gate (Figure 11a in PG), and pull-down (Figure 11a in PD) was designed to be 1:2:2 for high performance. Figure 11b shows the SNM curve, which is a representative FoM. SNM can be obtained as the largest possible square by superimposing the two voltage transfer curves of the CMOS inverter. In this study, SNM is defined as the length of the diagonal of a square [15]. If the external DC noise is greater than the SNM, the state of the SRAM cell is changed, and the data may be lost. In order to be tolerant to external DC noise, it is required that V_TH_, trip point and SNM are high. Also, the read voltage, which is the voltage level of the internal storage node where ‘0’ is stored, must be low during read access. The SNM of Case 1 with the highest V_TH_ and the smallest current flowing through the pass gate among the six cases was the highest. I_READ_ is the value of the current flowing through the pass gate in read mode, and the larger the I_READ_, the faster the read speed of the SRAM cell [16]. Therefore, the I_ON_ of Case 2 was the largest among the six cases, so I_READ_ was the largest. Lastly, BWRM is the voltage of the bit line when the data of the Q node and QB node in Figure 1 are changed in the write mode [17]. Even though the voltage of the bit line does not drop much, V(Q) and V(QB) are flipped when the BWRM increases. Conditions for write operation conflict with conditions for read operation. The lower trip point and the larger current flowing through the pass gate, it is easy to write the cell. Case 1, which has the highest read stability, has the smallest BWRM. Figure 11c shows the BWRM curves. The current flowing through the pass gate of Case 5 to which BO is applied is 13.3% higher than that in Case 1, to which BO is not applied, so BWRM is increased by 0.41%. The overall SRAM simulation results are listed in Table 3.

## 3. Conclusions

In this paper, optimization results for two options for reducing substrate leakage of 3 nm node mNS-FETs–the PTS doping scheme and the bottom isolation structure (BO)–are presented. If only PTS doping is adjusted without BO, the current and capacitance in the on-state will increase or decrease together, which creates a trade-off with regard to device performance. If BO is added, it is possible to reduce C_GGON_ by up to 11.1% compared to the former. In addition, the application of BO eliminates the source/drain-substrate junction, leading to a reduction of up to 78% in C_JDB_, resulting in additional device performance improvement. PTS doping requires a high concentration value of >5 × 10^18^ cm^−3^ regardless of the presence or absence of BO. Because the device characteristics of the mNS-FET are also related to the capacitance, performance verification is required through the operation of the dynamic circuit. In this study, the performance improvement according to PTS doping and BO was confirmed in logic and memory circuits. It was verified that in RO, which is a logic circuit, it is possible to increase the speed by 3.9–4.9% under iso-power conditions and decrease power by 10.8–12.6% under iso-speed conditions. In FA, another logic circuit, the power decreased by 1.3–15.3% and the delay decreased by 2.6–16.8% under the same static power condition. In the memory circuit SRAM, I_READ_ was the highest in Case 2, but when BO was applied, it showed uniform I_READ_ regardless of PTS doping. In the case of BWRM, stable BWRM can be obtained only when certain PTS doping is satisfied when BO is not applied. As a result, it is expected that the application of BO has the advantage of ensuring uniform device performance.

## Figures and Tables

**Table 1:** Geometrical parameters for multi-nanosheet channel FET.

Parameters	Values
Contacted poly-gate pitch *(CPP*)	45 nm
Gate length (*L_g_*)	16 nm
Inner spacer length (*L_sp_*)	6 nm
Channel thickness (*T_ch_*)	8 nm
Channel width (*W_ch_*)	30 nm
Channel oxide thickness (*T_ox_*)	0.3 nm
S/D length (*L_sd_*)	17 nm
Channel high-k thickness (*T_HK_*)	1.1 nm
Bottom oxide thickness (*T_BO_*)	20 nm
S/D over-etching depth (*T_bot_*)	8.5 nm
Channel doping	1 × 10^17^ cm^−3^
S/D doping	3 × 10^20^ cm^−3^
PTS doping (upper of substrate 1)	3.6 × 10^18^~1 × 10^19^ cm^−3^
Substrate 2 doping	1 × 10^17^ cm^−3^

**Table 2:** Performance comparison of mNS-FET 28T-FAs.

	w/o BO			w/BO		
Case type	Case 1	Case 2	Case 3	Case 4	Case 5	Case 6
Dynamic power [nW]	627.6	690.4	693.4	619.7	**587.1**	600.8
Delay [ps]	61.72	54.39	55.36	53.02	**51.33**	52
EDP [×10^−27^ J·s]	46.8	45.3	46.1	40.1	**36.8**	38.3

**Table 3:** Performance comparison of mNS-FET 6T-SRAMs.

	w/o BO			w/BO		
Case type	Case 1	Case 2	Case 3	Case 4	Case 5	Case 6
SNM [V]	**0.145**	0.14	0.141	0.142	0.141	0.141
I_READ_ [μA]	113.6	**134.8**	131.1	128.7	128.7	129.5
BWRM [V]	0.217	0.242	**0.244**	0.243	0.243	0.243

## Data Availability

Not applicable.
